# Relationship Between Genetic and Phenotypic Variations in Natural Populations of Perennial and Biennial Sagebrush

**DOI:** 10.1002/ece3.70419

**Published:** 2024-10-17

**Authors:** Khurelpurev Oyundelger, Lisa Großmann, Veit Herklotz, Dörte Harpke, Oyuntsetseg Batlai, Karsten Wesche, Christiane M. Ritz

**Affiliations:** ^1^ Department of Botany Senckenberg Museum of Natural History Görlitz Görlitz Germany; ^2^ General Botany, Insitute of Biochemistry and Biology, University of Potsdam Potsdam Germany; ^3^ Leibniz Institute of Plant Genetics and Crop Plant Research Seeland Germany; ^4^ Department of Biology, School of Arts and Sciences National University of Mongolia Ulaanbaatar Mongolia; ^5^ Chair of Biodiversity of Higher Plants, International Institute Zittau Technische Universität Dresden Zittau Germany; ^6^ German Centre for Integrative Biodiversity Research (iDiv) Halle‐Jena‐Leipzig Leipzig Germany

**Keywords:** *Artemisia*, genotype and phenotype, growth form, interrelationship between variations of genetic and functional traits with environment

## Abstract

Plant responses to environmental heterogeneity depend on life‐history traits, which could relate to phenotypical and genetic characteristics. To elucidate this relationship, we examined the variation in population genetics and functional traits of short‐ and long‐lived *Artemisia* species that are co‐occurring in the steppes of Mongolia. Mongolian steppes represent stressful and water‐limited habitats, demanding phenotypic modifications in the short term and/or genetic adaptation in the long term. However, detailed knowledge is missing about both plant phenotypic and genetic differentiation, and their interrelationships in temperate grasslands. Here, we investigated 21 populations of the widely distributed subshrub *Artemisia frigida* and the herbaceous biennial *Artemisia scoparia*. Genetic variation was assessed with newly developed simple sequence repeats (SSRs) markers. Functional trait data were collected from each individual, and data on environmental variables was collected for each population. We detected significantly higher genetic diversity in the biennial species (*H*_E_ = 0.86) compared with the perennial (*H*_E_ = 0.79). For both species, the largest share of genetic variation was partitioned within populations (96%). Population genetic structure in the biennial *A. scoparia* was weak, while the perennial *A. frigida* showed some spatial genetic structure, which was impacted by geographical factors, soil nutrients, and precipitation amount. Morphology‐related functional traits (i.e., plant height) were predominantly associated with environmental variables rather than with genetic variation, whereas physiology‐related trait (i.e., specific leaf area [SLA]) was partly genetically determined.

## Graphical abstract

The present study investigated the relationship between variations in population genetics and functional traits of short‐ and long‐lived *Artemisia* species that are co‐occurring in the steppes of Mongolia. We covered large spatial extent and collected numerous environmental variables (climate, geography, vegetation, and soil) to contrast the association between variations of functional traits and genetic diversity of the chosen species.

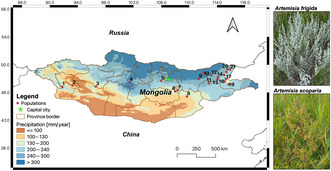

## 1 Introduction

It is widely acknowledged that a species’ genetic diversity and its variation are associated with life‐history traits, such as life form, breeding system, seed dispersal mechanism, and geographical range (Hamrick and Godt 1996; Nybom and Bartish 2000; Reisch and Bernhardt‐Römermann 2014). Species with outcrossing and mixed‐mating systems tend to have higher levels of genetic variation than selfing species (Nybom 2004). Short‐lived, non‐woody, self‐compatible, and early‐successional species, i.e., annuals/biennials, are characterized by higher genetic variation between populations, but lower genetic variation within populations. In contrast, long‐lived, woody, outcrossing, and late‐successional species, i.e., many perennials, have higher genetic variation within populations (Reisch and Bernhardt‐Römermann 2014). However, comparative studies such as that of Heelemann et al. (2015) found lower within‐population variation in perennial *Eriocephalus africanus* L. than in the annual species *Hemimeris racemosa* (Houtt.) Merrill. A comparison of perennial and annual wild species of the genus *Oryza* L. discovered that perennial species had higher population level genetic diversity but less genetic variation among populations than annuals (Zhou et al. 2008).

Even within a species, plant phenotypic variation is often high. Functional trait plasticity related to morphology (e.g., plant height), (eco)physiology (e.g., SLA), and life history (e.g., flowering time and seed traits) was found to be under genetic control in some model plants (Locascio, Lucchin, and Varotto 2009; Hughes, Soppe, and Albani 2019). Several studies detected correlations between phenotypic traits (morphological and functional trait variation) and genetic variation (Waitt and Levin 1998; Karbstein, Tomasello, and Prinz 2019; Csilléry et al. 2020). In particular, Waitt and Levin (1998) presented a meta‐study demonstrating a positive correlation between the genetic and phenotypic character traits of 27 species. However, trait variation does not necessarily coincide with genetic variation, especially if the trait is completely plastic (Chevin and Hoffmann 2017). Plasticity, i.e., phenotypic modification, allows for long‐term adaptation to the local environment and/or short‐term (reversible) responses. However, how genetic diversity and intraspecific functional traits interact at the population level, particularly in natural environments, remains poorly understood.

*Artemisia* L. (sagebrush) is a large and diverse genus that comprises over 500 taxa of annuals/biennials, perennial herbs, and shrubs or subshrubs distributed across temperate regions of the northern hemisphere (Riggins and Seigler 2012). Many species are clearly wind‐pollinated; however, some indication of insect pollination was observed (colorful capitula and sticky pollen; Vallès and McArthur 2001). *Artemisia* spp. inhabits arid, semi‐arid, and mesic environments spanning deserts to tundras, and their range of phenotypic diversity is broad (morphological, (eco)physiological, and reproductive traits), as is their range of ploidy levels (2*n* = 16 or 18 up to 2*n* = 144; Sanz et al. 2008). Although the genus offers ample opportunities for comparison, studies on genetic diversity and life history traits are hardly available. Al‐Ajmi et al. (2021) compared seven species of *Artemisia* and found a positive interspecific correlation between similarities in genetic variation among species. However, we do not know of any study that addressed intraspecific variation in traits and genetic structures.

*Artemisia frigida* Willd. and *Artemisia scoparia* Waldst. & Kit. are both outbreeding and wind pollinated species (Vallès et al. 2011) with a range of phenotypic variations. In this study, we aimed to test the effects of the environment on genetic variation and genetic structure of the short‐lived biennial *A. scoparia* and the long‐lived subshrub *A. frigida*, which are co‐occurring in the steppes of Mongolia. The flora of Mongolia lists 103 native *Artemisia* species (Baasanmunkh et al. 2022), among which species growing in dry steppes and forest steppes are the most numerous. Mongolia has one of the world's largest steppes, covering 1.2 million km^2^ and being home to thousands of steppe species (Munkhzul et al. 2021; Baasanmunkh et al. 2022). The continuous plain steppe of Mongolia allows for sufficient genetic exchanges between plant populations, as shown by former studies on the perennial grass *Stipa glareosa* P.A.Smirn. (Oyundelger et al. 2020) and on *A. frigida* (Oyundelger et al. 2021, 2023). In these studies, we detected moderate genetic structuring, which was mostly attributed to the differences in climate and edaphic conditions of the local populations rather than the geographical distance. However, the present study covers an even larger area of Mongolia, ranging from the western Altai Mountains to the eastern Mongolian Steppes. Specifically, we aimed to answer the following questions: (i) How do genetic diversity and population structure differ between the two *Artemisia* species? (ii) Do environmental factors relate to the genetic variation of the species across the Mongolian steppe? (iii) Are functional traits related to genetic diversity and/or abiotic habitat heterogeneity?

## 2 Materials and Methods

### 2.1 Study Species: *Artemisia frigda* and *Artemisia scoparia*

**FIGURE 1:**
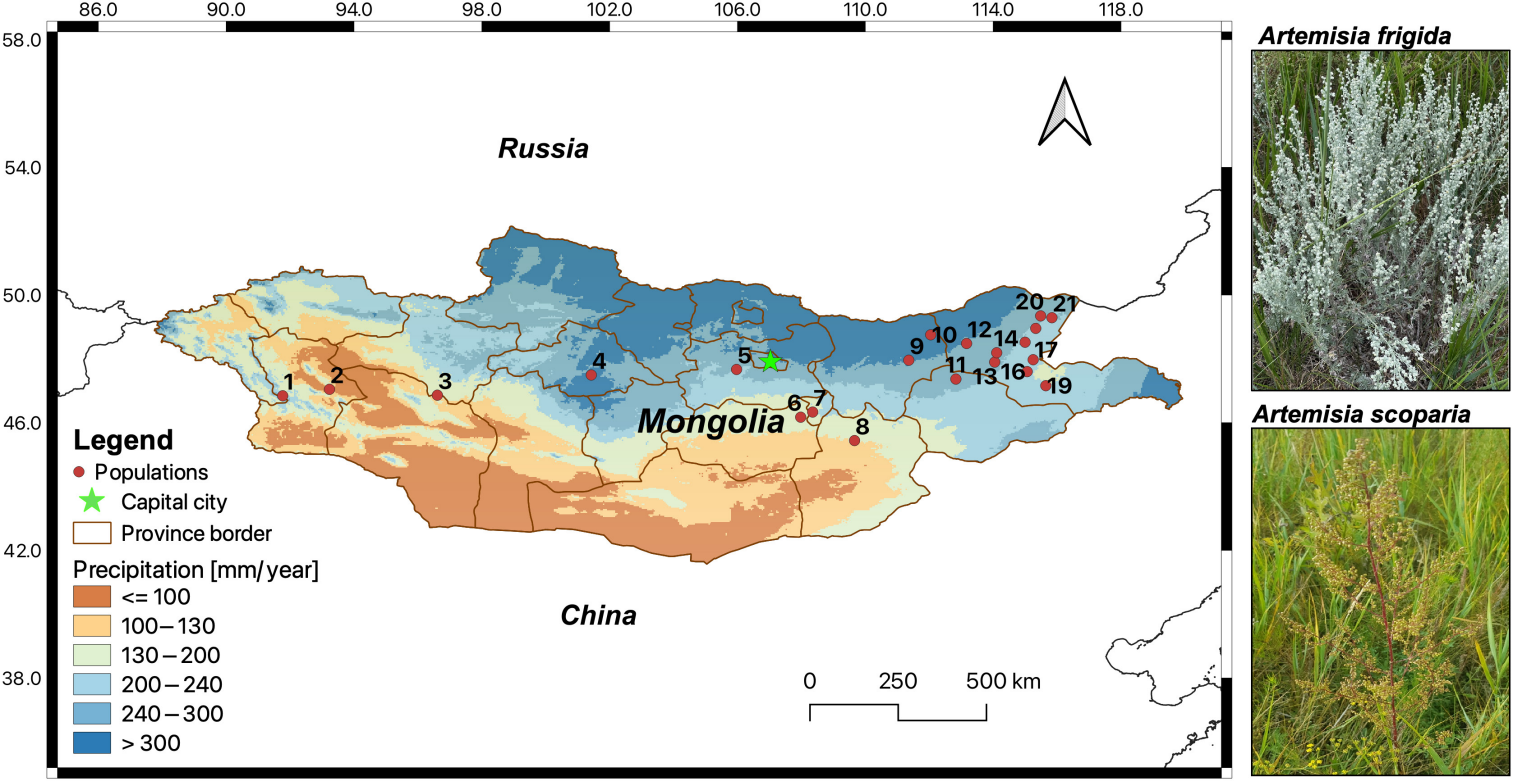
Study area with locations of 21 populations sampled for *Artemisia frigda* and *Artemisia scoparia* across Mongolia. Precipitation data were derived from Fick and Hijmans (2017).

Perennial prairie‐sage (*A. frigida*) has the largest natural range within its genus, being distributed across the North American prairie and the Eurasian steppe, whereas *A. scoparia* is a biennial species widely distributed from Central Europe to East Asia. Species’ ranges overlap in Inner Asia and specifically in Mongolia, where they are common steppe plants (Hilbig 1995). They share the same breeding system (outbreeding) and dispersal mechanism (wind), yet differ in their life form (biennial herb vs. perennial subshrub). The perennial *A. frigida* grows primarily in mountains, hillsides, and ruderal sites in steppes (Tkach et al. 2008). It bears a dense silvery pubescence and has woody ascending stems that are usually strongly branched (Figure 1). The biennial *A. scoparia* is found in riverbanks, as well as in ruderal sites in steppes and semi‐deserts. Its stems are initially pubescent, becoming glabrous and strongly branched in the middle and upper parts (Figure 1). *Artemisia frigida* and *A. scoparia* are pioneer plants at grazing disturbed sites and also occur in the early recovery stages of abandoned land that underwent severe soil erosion (Jiao et al. 2013; Wang et al. 2022). Both species have high seed yields with small and light seeds (*A. frigida*: 0.106 g and *A. scoparia*: 0.047 g) that are easily propagated by wind and are then buried into soils (Yi et al. 2019).

*Artemisia scoparia* belongs to the subgenus *Dracunculus* Besser representing the most basal lineage of *Artemisia* (clade divergence in 17.6 ± 2.1 Mya), while *A. frigida* is part of the subgenus *Absinthium* DC. (clade node 6.8 ± 0.8 Mya; Sanz et al. 2011; Hussain et al. 2019). *Artemisia frigida* comprises diploids (2*n* = 2*x* = 16) as well as tetraploids (2*n* = 4*x* = 36; Pellicer et al. 2010; Korobkov, Kotseruba, and Probatova 2014). In *A. scoparia*, mostly diploid cytotypes were observed (2*n* = 2*x* = 16 or 18; Pellicer et al. 2010); yet 2*n* = 4*x* = 32 or 36 have also been reported from Slovenia, Siberia, and recently from the Western Himalayas (Kawatani 1964; Amelchenko 1979; Gupta, Goyal, and Singh 2014).

### 2.2 Study Design and Sampling

Sampling was carried out along a broad‐scale longitudinal precipitation gradient from western to eastern Mongolia during the summers of 2018 and 2019 (Figure 1). Fresh leaf materials were collected from 21 populations where both species co‐occurred. For each population, representative herbarium specimens were deposited at Herbarium Senckenbergianum Görlitz (GLM). As a result, we sampled thirteen eastern (E) populations and four western (W) and four central (C) populations across various steppe vegetation types (Table 1).

**TABLE 1:** Characteristics of the study sites: population code, localities, main climatic variables, steppe vegetation type and region.

Pop code	Locality and province	Longitude	Latitude	Altitude (m)	MAT (°C)	MAP (mm)	Summer temp. (°C)	Summer prec. (mm)	cvP (%)	Steppe type	Region
1	Munkhkhairkhan, Khovd	91.765	46.841	1781	−6.1	147	8.9	87	33	MoS	W
2	Center of Khovd, Khovd	93.228	47.044	1355	2.6	115	19.5	77	42	DrS	W
3	Taishir Soum, Govi‐Altai	96.605	46.860	2009	−1.6	172	14.7	105	29	DrS	W
4	Khotont Soum, Arkhangai	101.421	47.492	1608	−1.3	300	14.3	198	24	MoS	W
5	Hustai National Park, Tuv	105.968	47.666	1264	0.9	167	18.3	118	24	MoS	C
6	Tsagaandelger, Dundgovi	107.975	46.170	1280	2.2	117	19.9	82	42	DrS	C
7	Choir, Dundgovi	108.350	46.331	1270	1.9	135	19.7	92	40	DrS	C
8	Altanshiree, Dundgovi	109.660	45.438	1007	3.8	126	21.9	84	31	DeS	C
9	Batnorov, Khentii	111.357	47.955	1078	0.5	286	18.8	194	25	DrS	E
10	Norovlin, Dornod	112.044	48.751	1020	0.6	277	18.6	192	29	DrS	E
11	Hulunbuir, Khentii	112.831	47.364	1008	0.4	231	18.6	164	37	DrS	E
12	Tsagaan‐Ovoo, Dornod	113.167	48.480	1009	1.4	240	19.6	166	34	DrS	E
13	Bulgan, Dornod	114.046	47.896	961	1.5	238	19.9	163	46	DrS	E
14	Bayantumen, Dornod	114.109	48.190	991	1.4	210	19.7	142	44	DrS	E
15	Choibalsan, Dornod	114.997	48.519	847	1.5	209	20.3	141	49	DrS	E
16	Matad, Dornod	115.062	47.598	761	2.1	184	20.5	130	52	DrS	E
17	Matad, Dornod	115.250	47.971	1075	1.4	198	19.9	139	53	DrS	E
18	Choibalsan, Dornod	115.331	48.953	909	1.0	231	19.9	153	46	DrS	E
19	Shar‐Khudag, Dornod	115.646	47.157	1011	1.5	199	19.7	144	52	DrS	E
20	64n toochig, Dornod	115.485	49.344	650	1.3	232	20.4	154	45	DrS	E
21	Otor pasture, Dornod	115.837	49.288	821	1.1	239	20.3	159	44	DrS	E

Abbreviations: C, central; cvP, coefficient of variation of interannual precipitation; DeS, desert steppe; DrS, dry steppe; E, eastern region of Mongolia, coordinates are in WGS84; MAP, mean annual precipitation; MAT, mean annual temperature; MoS, mountain steppe; Summer prec., summer mean annual precipitation; Summer temp., summer mean annual temperature; W, western.

At each site, 15 individuals per species were sampled within a 10 m × 10 m plot. Within these plots, plant community composition and total cover (%) of vascular plants were recorded, and a sample of topsoil (1 – 5 cm depth) with fine plant roots and the humic layer was collected. Soil samples were separated from litter, debris, and after shifting through a 2 mm sieve the following measurements were conducted in the laboratory: pH value, electrical conductivity (EC, as a proxy for salinity), plant available P, N%, organic C%, and C/N ratio. All results refer to oven‐dried soil (75°C, 18 h). Moreover, plots were classified into different steppe types according to “The steppe vegetation of Mongolia” (Tuvshintogtokh 2014) based on our sampling location, which was also validated by our field‐based plant community composition data.

Three functional traits were measured in the same individuals sampled for molecular data. In the field, “height of inflorescence (HI)” (if plants were flowering), “height of vegetative part (HV),” and leaf area for the trait “SLA” were measured. The HI was determined as the height from ground level to the tip of the highest inflorescence and the HV as the height of one randomly selected vegetative branch per plant. In *A. frigida*, vegetative and generative shoots differ in length. Thus, both heights were chosen as traits. *Artemisia scoparia* does not develop sterile shoots, and thus only the HI was applicable. For SLA, two fresh leaves were taken from each individual (30 leaves per site) and scanned using a Conrad P‐573 handheld document scanner. Scanned pictures were later analyzed with ImageJ (Abràmoff, Magalhães, and Ram 2004) to determine the leaf area. Leaves were then air‐dried for more than a month, and biomass weight was measured with a Mettler Toledo XP6 balance in the laboratory. The SLA was then calculated by dividing leaf area by dry mass (Perez‐Harguindeguy et al. 2013). Population‐level trait data and their correlation matrices, indicating their independence, are provided in Table S1.

Meteorological data of 20 years (mean annual temperature [MAT], mean annual precipitation [MAP], and mean spring temperature [March–May], mean summer temperature, and mean summer precipitation [June–August] between 1994 and 2013) were retrieved for each locality from the high‐resolution CHELSA_V1 dataset, which has the advantage of capture interannual precipitation variation (Karger et al. 2017). The coefficient of interannual variation of annual precipitation (cvP) was estimated based on the retrieved MAP data and was also used as a predictor since cvP is a critical driver of rangeland dynamics (von Wehrden et al. 2012).

### 2.3 Molecular Analyses and Microsatellite Marker Development

Two randomly selected individuals of each species from two distinct populations were used to develop new SSR markers by applying whole genome sequencing (WGS). A previous study by Oyundelger et al. (2021), gives detailed steps for DNA extraction, library preparation, quality control, and bioinformatics in SSR development. Raw sequencing data were submitted to the NCBI Sequence Read Archive (SRA) and made publicly accessible under BioProject: PRJNA680535.

A total of 20 and 21 SSR markers were then tested for optimization in *A. frigida* and *A. scoparia*, respectively, using randomly selected samples from more than ten populations containing 8–16 samples. Furthermore, cross‐checking of markers for both species was performed, and ten SSR markers published for *A. frigida* in the master thesis of Wang (2011) were tested with our samples in parallel. Based on reproducibility and polymorphism, 11 markers were chosen for each species. Detailed information on SSR markers of *A. frigida* can be found from Oyundelger et al. (2021). Information about species‐specific SSR markers for *A. scoparia* developed for this study are presented in Table 2. Amplifications of a total of 22 SSR markers were performed in a volume of 12.5 μL, and customized PCR reaction mixtures and cycling programs were used (see PCR details from Table S2). Individuals of all 21 populations from both species exhibited a maximum of four alleles per locus, indicating prevailing tetraploidy (see Table S3 for ploidy information).

**TABLE 2:** Characterization of eleven polymorphic microsatellite markers used in this study for *Artemisia scoparia*.

No.	Locus	Repeat motif	Primer sequences (5′–3′)	*T*_a_ (°C)	Allele size range (bp)	Fluorescent dye	PCR type
1	*Arcs2*	(GT)9	F: TGTAAAACGACGGCCAGTTCTCCTTTCTGATTCATTGG	55	585–620	6 FAM	Multiplex
			R: CGAGATGAATTTGCGTCAT				
2	*Arsc12*	(TGT)9	F: TGTAAAACGACGGCCAGTGGACATTTGAATGATGTTCG	55	200–265	6 FAM	
			R: AAGTCTTCCGCCAGCTATA				
3	*Arsc7*	(TG)11	F: TGTAAAACGACGGCCAGTTGT CCATCAAGATACCTATGC	55	520–560	VIC	Multiplex
			GGTTATCGCCTCTCATTTG				
4	*Arsc11*	(ACA)8	F: TGTAAAACGACGGCCAGTGAACGGGAAGATTACAAGC	55	130–180	VIC	
			R: CACCAATATTACCTGGTGTG				
5	*Arsc18*	(ATG)8	F: TGTAAAACGACGGCCAGTACACTGGAAAGCTATGTGC	55	610–660	PET	Multiplex
			R: CGAGTCACAGTCATGGTC				
6	*Arsc19*	(TGA)8	F: TGTAAAACGACGGCCAGTCCT CAAACCTTGAAAGATAGC	55	350–400	PET	
			R: CCGTATGAGTTAAGCAATCAG				
7	*Arsc17*	(TGA)8	F: TGTAAAACGACGGCCAGTAATGGATTATGTTGATAGCCA	55	135–160	6 FAM	Singleplex
			R: CAAGTTCCGTTGACTCG				
8	*Arsc14*	(ATA)8	F: TGTAAAACGACGGCCAGTATG CACATAATATCCGAGC	55	270–325	VIC	Singleplex
			R: GTGCTGAGACCGAATGC				
9	*Arsc20*	(ACA)14	F: TGTAAAACGACGGCCAGTGAC ACCCATAGACAGGAGC	55	~500	NED	Singleplex
			R: GTCAGCTCGAAGCTTTCC				
10	*Arsc21*	(TGT)8	F: TGTAAAACGACGGCCAGTTGC CTTTGCAACAATTAAC	55	110–128	NED	Singleplex
			R: GCTGCAAACATTACGTAAGC				
11	*Ch468*	NA	F: TGTAAAACGACGGCCAGTTAG GGTTGCAGAAGATAAAC	55	160–236	PET	Singleplex
			R: GCTTCTTCACTTCCTACTAAAG				

*Note:* Details on SSR markers for *Artemisia frigida* can be found in Oyundelger et al. (2021).

### 2.4 Statical Analyses

#### 2.4.1 Analysis of Genetic Diversity and Population Structure

To compare the genetic diversity within each species, we employed two programs, which allowed handling of microsatellite data for polyploids and species with mixed ploidy: GenoDive v.3.04 (Meirmans 2020) and the R‐package *Polysat* v. 1.7 (Clark and Jasieniuk 2011) in R v.4.0.3 (R Core Team 2020). Estimators of genetic diversity comprised allelic diversity (AD), percentage of polymorphic loci (PPL), observed heterozygosity (*H*_O_), expected heterozygosity (*H*_E_), and inbreeding coefficient (*G*_IS_), all of which were calculated using GenoDive. Bruvo distances were computed with the R‐package *Polysat* v.1.7 (Bruvo et al. 2004). Using the R‐package *vegan* (Oksanen et al. 2007), we calculated the mean Bruvo distance among individuals for any given population (hereafter ‘Bruvo index’; see detail in Oyundelger et al. 2021), which was then used as a surrogate for genetic diversity (see Table S4 for the genetic diversity indices). A paired *T*‐test was used to determine the significance of the difference in genetic diversity indices between two species.

Coefficients of genetic differentiation (*F*_ST_ and *G*_ST_) were estimated using *Polysat* (Table S5). Population genetic structure was further analyzed with principal coordinate analysis (PCoA) using population‐wise *F*_ST_ distance using the R‐package *ape* (Paradis and Schliep 2019). In order to reveal environmental variables that were significantly associated with population genetic structure of the species, environmental and vegetation variables were fitted post hoc on the ordination using *vegan*, and plots were visualized with *ggplot2* (Wickham 2011).

To examine the partitioning of genetic variation between and within populations, analysis of molecular variance (AMOVA; Excoffier, Smouse, and Quattro 1992) was performed in R‐package *poppr* (Kamvar, Tabima, and Grünwald 2014) based on the individual‐level Bruvo distance matrix estimated with *Polysat*.

#### 2.4.2 Relationship Between Genetic and Spatial Distances

To assess the overall relationship between genetic and spatial distances, Mantel tests between genetic distance (linearized population level pairwise *F*_ST_ (*F*_ST_/(1 − *F*_ST_))) and geographic distances (Euclidean distances) were computed through 10000 randomizations using the R‐package *vegan* (Oksanen et al. 2007). Further Mantel tests were then conducted between genetic distances and (a) climatic differences (Euclidean distance of centred and standardized climatic variables); (b) distance of soil indicator variables (Euclidean distance of centred and standardized variables), and (c) differences in plant community composition (Bray‐Curtis's distance based on log‐transformed species' cover).

#### 2.4.3 Relationships of Functional Trait Variation With Genetic and Environmental Patterns

We estimated population‐level means and coefficients of variation (CV) for trait variables, the latter as the ratio of standard deviation to mean. We checked collinearity among traits (mean and CV) with Pearson's coefficient (Table S1) using the R‐package *corrplot* (Wei et al. 2021). As correlation coefficient values (*r*) of the mean and CVs were below ~|0.7|, we did not exclude particular functional traits.

To assess whether functional traits are related to environmental heterogeneity and genetic diversity, we fitted linear models (Dobson and Barnett 2018) with mean and CV of traits as the dependent variables. We again used *corrplot* for an exploratory analysis of associations among measures of genetic diversity. As a result, *H*_E_ was chosen as the main response variable, as it had the highest correlation and depends less on population history (e.g., bottlenecks) compared to the other indices (Rosenberg 2004; Szczecińska et al. 2016). For the predictors, we first checked correlations among environmental variables to select representative variables based on their importance and independencies (*r* < |0.7|; see Table S6 for the data and their correlations). As a result: MAP, MAT, and cvP for climate; altitude for topography, and soil C/N ratio for soil nutrient contents were initially used as predictors for the models.

All predictors were first scaled to zero mean—unit variance (*z*‐scores) to make effect sizes comparable. The response variable: cvIH of *A. frigida* was log‐transformed due to its non‐normal distribution; other response variables (cv and means) were in normal distribution, and thus no transformation was done. We then conducted model simplification by dropping the least relevant variables from linear models until a null model with intercept only. Models were compared using ANOVA, the summary was used to estimate significance and to choose the most parsimonious models. Lastly, plotting was used to check residuals of the models for possible deviations from normality and reasonable distribution of variances.

## 3 Results

### 3.1 Comparison of Genetic Diversity Between the Perennial and Biennial *Artemisia*

**FIGURE 2:**
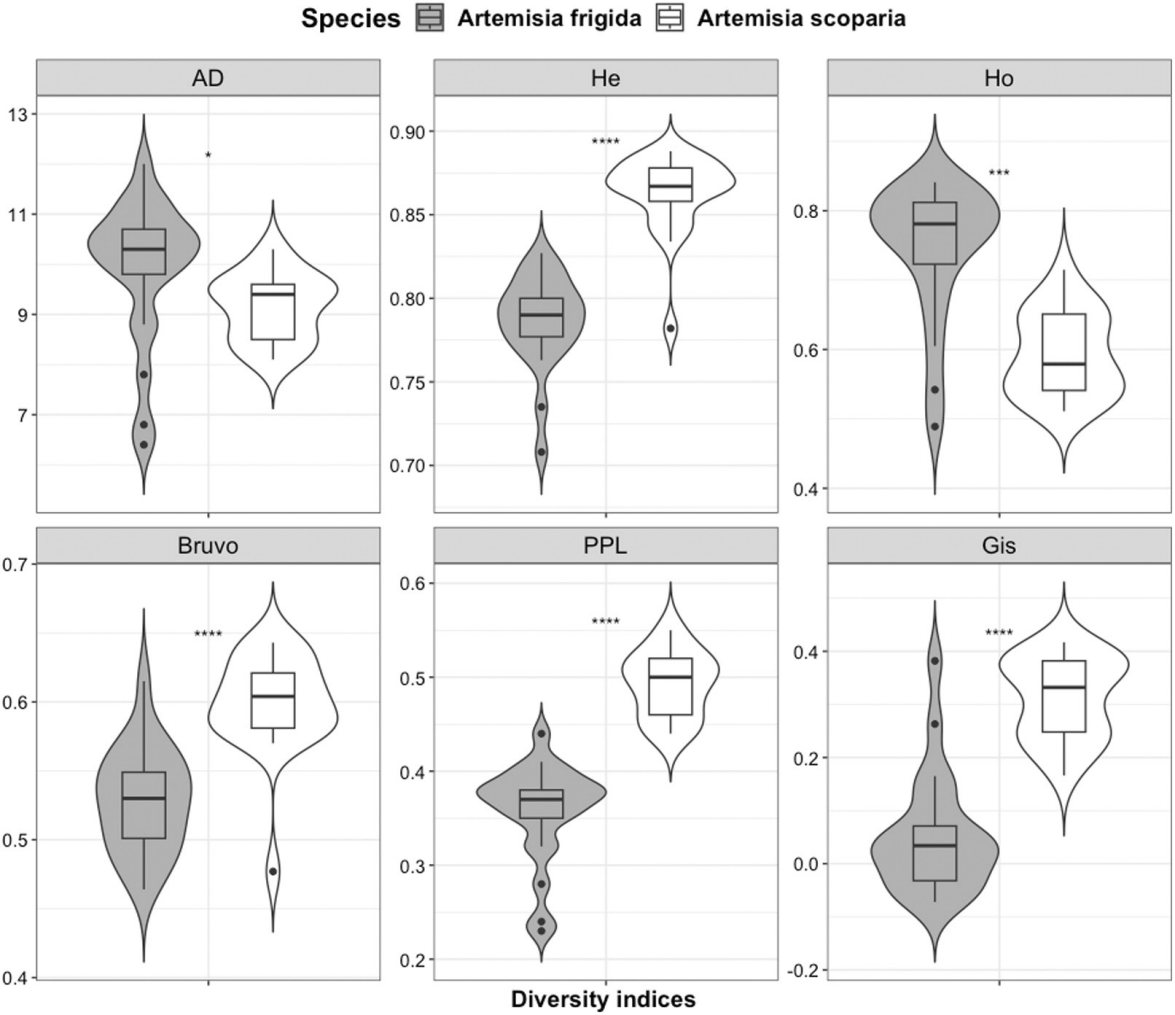
Violin boxplots of genetic diversity indices of the perennial *Artemisia frigida* (*N* = 304) and the biennial *Artemisia scoparia* (*N* = 303) (AD, allelic diversity; Bruvo, Bruvo index; *G*_IS_, inbreeding coefficient; *H*_E_, expected heterozygosity; *H*_O_, observed heterozygosity; PPL, percentage of polymorphic loci). Significance codes: ****p ≤ 0.0001; ***p < 0.001; *p < 0.05.

The overall polymorphic information content (PIC) of newly developed species‐specific SSR markers was high (PIC = 0.77 and 0.84) for both *A. frigida* and *A. scoparia*. Paired *T*‐test revealed that proxies of genetic diversity differed between two the *Artemisia* species (Figure 2). Specifically, *H*_E_, Bruvo, PPL, and *G*_IS_ of the biennial *A. scoparia* were significantly higher than in the perennial *A. frigida*. In contrast, AD and *H*_O_ were larger in the perennial than the annual species, yet with lower significance. Details for estimators of genetic diversity are presented in Table S4.

### 3.2 Population Genetic Variation and Relationship With Environmental Variables

**FIGURE 3:**
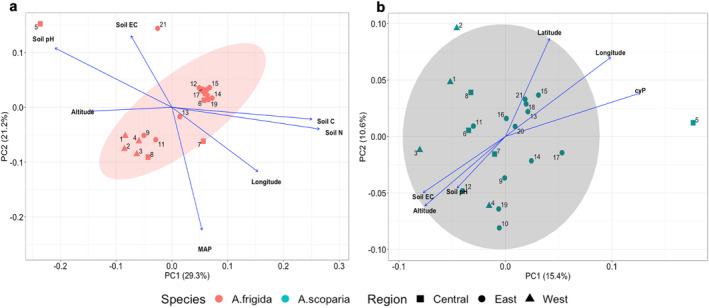
Principal coordinate analyses (PCoA) based on *F*_ST_ distances of the (a) perennial *Artemisia frigida* and (b) the biennial *Artemisia scoparia* among 21 populations across three regions (east, central, and west) of Mongolia. Each symbol represents one population, and 95% confidence intervals are indicated by shaded area. Environmental predictors were fitted post hoc on the ordination plot (only those that passed *p* < 0.05 according to a test with 1,000 permutations are shown). Result of *post hoc* analyses indicating the importance of environmental variables are provided in Table S8.

Coefficients of genetic differentiation of the two species across 21 populations were low overall, suggesting that isolation is at most moderate over the distances considered here. However, population differentiation of *A. frigida* was slightly more pronounced (Global *F*_ST_ = 0.078 and Global *G*_ST_ = 0.071) than of *A. scoparia* (Global *F*_ST_ = 0.064 and Global *G*_ST_ = 0.055). The most genetically distant population was population 5 (Hustai National Park) in both species (dissimilarity data provided in Table S5). Analysis of molecular variance showed that in both species, the highest genetic variation resided between individuals, while genetic variation partitioned among regions was slightly higher in *A. frigida* than *A. scoparia* (0.97% and 0.83%, respectively; Table 3). The ordination plots suggested that there was no pronounced genetic differentiation among steppe types and regions of Mongolia (individual level PCoA in Table S7), although *A. frigida* exhibited some population level genetic structure (Figure 3). In the PCoA ordination of *A. frigida*, the first two axes explained about 50% of the genetic variation, and some structuring of eastern vs. western populations mixed with central populations was discernible. According to post hoc fitting of predictor variables, longitude, altitude, mean annual precipitation (MAP), soil carbon, nitrogen, pH, and soil electrical conductivity (EC) showed a significant association with genetic structure (Figure 3a). In total, 26% of the total genetic variation was explained by the first two axes in the populations of *A. scoparia*, representing more continuous patterns among populations. Main structures along axis 1 and 2 were significantly correlated with altitude, soil pH, and EC together with longitude, latitude, and coefficient of variation of interannual precipitation (cvP), with western populations being in the upper left (Figure 3b). The ordinations demonstrated that soil pH and EC, as well as soil C and N, exhibit covariance, as proven by their high correlations (*r* = 0.78 and *r* = 0.99; Table S6). Results of *post hoc* fitting predictor variables on the PCoA are provided in the Table S8.

**TABLE 3:** Summary of analysis of molecular variance (AMOVA) of the perennial *Artemisia frigida* and the biennial *Artemisia scoparia* of 21 populations across Mongolia.

Source of variance	df	Sum sq	Variance component	% Total	Φ statistic
*Artemisia frigida*					
Between regions	2	12.56	0.028	0.97***	0.037
Between populations	18	72.03	0.080	2.69***	0.027
Within populations	283	806.44	2.850	96.34***	0.009
Total	303	891.03	2.958		
*Artemisia scoparia*					
Between regions	2	2.21	0.004	0.83***	0.035
Between populations	18	11.25	0.013	2.69***	0.027
Within populations	282	128.68	0.456	96.47***	0.008
Total	302	142.14	0.473		

Abbreviations: % total, percentage of variation; df, degrees of freedom; Sum Sq, sum of square.

*** *p* ≤ 0.0001.

The Mantel tests on association between genetic structures (linearized *F*_ST_—*F*_ST_/(1 − *F*_ST_)) with various environmental variable distances revealed an overall negligible relationship with the genetic distances in both species (Table S9). Geographic distance and the distance of soil nutrient values in particular showed a significant but weak correlation with the genetic distance of *A. frigida* (*r* ^2^ = 0.05*** and *r* ^2^ = 0.02*). In contrast, *A. scoparia* did not exhibit an isolation by distance effect, while a weak correlation with climatic distance was observed.

### 3.3 Associations of Functional Traits With Genetic and Environmental Variations

Results of linear models showed that means as well as coefficients of variation of functional traits in *A. frigida* were associated with climatic and geographic variables, whereas in *A. scoparia* genetic diversity and soil nutrients had a significant relationship with SLA (Table 4). In the perennial *A. frigida*, altitude was positively associated with the physiology‐related trait (mean SLA), while variations of morphology‐related traits, cvHI and cvVH, were significantly affected by MAT, MAP, and cvP. In the biennial *A. scoparia*, genetic diversity showed an association with mean SLA and soil nutrient contents with the variation of SLA. With the exception of altitude and cvP, all significant associations were negative (scatter plots with linear regression lines of the significant models are provided Tables S10 and S11).

**TABLE 4:** Summary of the retained parsimonious and significant linear models assessing the associations of functional traits of *Artemisia frigida* and *Artemisia scoapria* with the genetic diversity and environmental variables.

	Functional traits	Predictor	Estimate	Std. error	Pr(>|*t*|)
*A. frigida*	Mean SLA	(Intercept)	0.15	0.006	< 0.001***
		Altitude	0.02	0.006	0.012*
	CV height of inflorescence	(Intercept)	1.29	0.022	< 0.001***
		MAP	−0.09	0.023	0.002**
		MAT	−0.08	0.025	0.005**
		cvP	0.07	0.025	0.01**
	CV height of vegetative part	(Intercept)	31.78	1.652	< 0.001***
		MAT	−3.86	1.693	0.034*
*A. scoparia*	Mean SLA	(Intercept)	0.15	0.007	< 0.001***
		*H*_E_	−0.03	0.007	0.001***
	CV SLA	(Intercept)	1.61	0.037	< 0.001***
		Soil C/N	−9.06	3.602	0.021*

*Note:* Pr(>|*t*|) – significance *p*‐value. Significance codes: ***p ≤ 0.001; **p < 0.01; *p ≤ 0.05.

## 4 Discussion

### 4.1 Population Genetic Diversity and Differentiation of *Artemisia frigida* and *Artemisia scoapria*

Life form and breeding system of plants are known to have a major influence on species' genetic diversity and population genetic structure (see Nybom and Bartish 2000; Reisch and Bernhardt‐Römermann 2014; De Kort et al. 2021). Our chosen *Artemisia* species both have a wide range of distribution, are wind/water dispersed, outcrossing, and showed prevailing tetraploid cytotypes, making a direct comparison of diversity indices possible. Population‐level mean values of the genetic diversity in both *Artemisia* species were higher (*A. frigida*: *H*_E_ = 0.79 and *A. scoapria*: *H*_E_ = 0.86) than in the review of Nybom (2004) for similar life history traits. The genetic diversity was significantly higher in the biennial *A. scoparia* than in the perennial species, according to four of the six diversity indices (*H*_E_, *G*_IS_, Bruvo, and PPL; Figure 2). This is in line with the study of Balfourier, Charmet, and Ravel (1998), who compared outcrossing annual and perennial ryegrass (*Lolium* L.) species. Probably, the effective population size and recombination rate are higher in the biennial than in the perennial. In short‐lived species, recombination rate is higher as a result of their shorter life cycles and smaller genome/lower DNA content (Brazier and Glémin 2022), which may lead to a higher level of genetic diversity. Indeed, Garcia et al. (2004) reported that genome size of *A. scoparia* was the smallest (1C = 1.77 pg) within the studied species, while the genome size of *A. frigida* was 2.63 pg. Furthermore, in *A. frigida*, a smaller number of plants may participate in reproduction, as it is often subject to intensive grazing in natural and permanent pastures, and some individuals may survive vegetatively over several seasons. However, this observation is in contrast to some review studies that compared the genetic diversity of different life forms, utilizing allozyme and RAPD markers (see Hamrick and Godt 1990, 1996; Nybom and Bartish 2000; Nybom 2004) and AFLP markers (Balfourier, Charmet, and Ravel 1998; Reisch and Bernhardt‐Römermann 2014). Nonetheless, individual life history traits, as well as genetic markers and diversity indices utilized, affect estimates of population genetic diversity, making the direct comparisons among studies somewhat questionable.

Patterns of genetic variation in the two species did not differ much, with spatial differences (among regions) explaining about 1% of the genetic variation, while barely 2%–3% variation resided among populations, and the highest variation (more than 95%) was explained by within‐population variations (Table 3). Yet, the populations of the perennial *A. frigida* represented some structure illustrated in the PCoA, having fuzzy eastern and western clusters associated with altitude, longitude, amount of precipitation, and soil salinity (Figure 3a). Patterns in the biennial species were more continuous and impacted by geographical factors, like longitude, latitude, and altitude, as well as the coefficient of interannual precipitation variation (Figure 3b). Population 5 (Hustai NP) is a geographically central population that, however, represented the greatest genetic distance from others in both species (see PCoA; Figure 3 and Table S5 for differentiation matrices). This pattern has been seen in our former studies (see Oyundelger et al. 2021, 2023) and is now supported by the analysis of a second species, indicating this region has a distinct regime of gene flow and/or population connectivity, most likely due to its proximity to the local livestock trade center where animals from all over the country are brought in and may carry seeds.

Only few studies have compared the genetic variation of herbaceous species with different life forms (perennial vs. annual) in the same spatial context (Balfourier, Charmet, and Ravel 1998; Zhou et al. 2008; Heelemann et al. 2015), but their findings were contradictory: Zhou et al. (2008) found the highest molecular variation among populations in the annual (78%) than the perennial wild rice species (52%). While Balfourier, Charmet, and Ravel (1998) and Heelemann et al. (2015) reported that most of the total genetic variation was accounted for within populations in perennial (91%) and annual ryegrass (90%); and wild rosemary species (perennial: 89% and annual: 87%), respectively. Our result was in line with the latter, as within population variations were as high as 96% in both species. Furthermore, genetic variation between populations of the perennial was only marginally higher than that of annual species; yet both were comparably low. The low level of genetic variation between populations and regions, as well as weak correlations between genetic differences with environmental distances, indicates considerable historical and current gene flow between populations, supporting our former studies (Oyundelger et al. 2021, 2023).

### 4.2 Associations of Functional Traits With Genetic and Environmental Variations

Mean values as well as variations of morphology‐ (IH and VH) and (eco)physiology‐ (SLA) related traits were predominantly associated with environmental variables rather than with genetic variation (Table 4). This indicates that the traits showed substantial plasticity in response to environmental differences, as demonstrated by a number of other studies (see Gratani 2014; Chevin and Hoffmann 2017; Matesanz and Ramírez‐Valiente 2019). Specifically, climate (MAP, MAT, and cvP) was found to be the most important factor influencing the morphological trait variations of the perennial *Artemisia*. This, of course, indicates the importance of climatic conditions for plant growth, as has been previously shown for plant species occurrence and abundance in the Mongolian steppe (von Wehrden and Wesche 2007; von Wehrden et al. 2010). In *A. frigida*, morphological differentiation is probably promoted by site‐dependent microhabitat differences, primarily in temperature and water availability. Morphological differences become even more pronounced, particularly due to the harsh climate in steppes (MAT: min (−6.1) to max +3.8°C) with overall limited water availability (MAP: min 117 mm to max 300 mm), as demonstrated by our linear model (Table 4). Phenotypic differences were pronounced between sites/populations, whereas genetic differentiation was less evident (Global *F*_ST_ = 0.064). This is in line with a large body of literature showing plant phenotypic trait responses and genetic differentiation patterns varying highly in abiotic and biotic environmental conditions (Odat, Jetschke, and Hellwig 2004; Bucher et al. 2016; König et al. 2018), and plant trait differentiations being even enhanced in extreme environments (Chevin and Hoffmann 2017; Karbstein, Tomasello, and Prinz 2019).

SLA relates to photosynthesis, relative growth rate, and stress tolerance (Perez‐Harguindeguy et al. 2013) and is known to be subject to substantial plasticity (Pan et al. 2013; Stotz et al. 2022) as well as being partly under genetic control (Knight and Ackerly 2003; Scheepens, Frei, and Stöcklin 2010). In our study, mean SLA was significantly associated with altitude in *A. frigida* and with genetic diversity in *A. scoparia*. Soil nutrient availability also had a significant impact on the variation of the SLA in *A. scoparia*, supporting the common observations, as we detected the effect of both environment and genetics on SLA (Table 4). Significant relationships of the mean and cvSLA with environmental variables were observed in other studies. For instance, Woodward (1983) noted a negative association between altitude and SLA in *Festuca* L. and *Carex* L. species, which was explained by an underlying relationship between altitude and temperature. Yulin et al. (2005) detected an increasing SLA in habitats with higher amounts of soil nutrients (total nitrogen and organic carbon) in *Artemisia halodendron* Turcz. ex Besser, as soil nutrient stress is a major limiting factor for plant growth. A global study has shown a positive association between soil fertility and SLA, whereas negative relationships exist between soil C/N ratio and SLA (Ordoñez et al. 2009), supporting our findings. Furthermore, genetic effects on SLA variance were observed in *Campanula* L. (Scheepens, Frei, and Stöcklin 2010), which were attributed to selection‐induced adaptations. The same may hold true for our observation that genetically less diverse populations represented a larger mean SLA, as a result of local adaptation. Yet, this negative association might be rather an artifact attributed to the (natural outlier) population 5 (Hustai NP), where the lowest population‐level diversity (*H*_E_ = 0.78) and the largest mean SLA (0.24 mm/mg) were detected (see relationship in Table S10).

## 5 Conclusion

Understanding plant adaptation—both in terms of morphological and genetic aspects—to environmental heterogeneity has been a focal point of many studies. However, steppe plants have rarely been investigated, and no comparative studies of species with different life‐history traits have been conducted to date. Our findings demonstrated that genetic diversity in both species was relatively high (*A. frigida*: *H*_E_ = 0.79 and *A. scoparia*: *H*_E_ = 0.86), and their genetic variation and functional trait characteristics were significantly affected by geographical factors and soil nutrient contents. Surprisingly, climatic factors exhibited a relatively limited impact, and when there was an effect, it was primarily associated with the amount and variation of precipitation. This aligns with the overarching observation in Mongolia that precipitation serves as the primary limiting factor for plant growth, occurrence, and abundance. Thus, plants in these areas require significant adaptations to thrive in the water‐limiting habitats while retaining sufficient genetic diversity.

## Author Contributions

**Khurelpurev Oyundelger:** data curation (equal), formal analysis (lead), investigation (lead), methodology (lead), project administration (supporting), validation (equal), visualization (lead), writing – original draft (lead), writing – review and editing (lead). **Lisa Großmann:** formal analysis (supporting), investigation (supporting), methodology (supporting), visualization (supporting), writing – review and editing (supporting). **Veit Herklotz:** data curation (supporting), investigation (equal), methodology (equal), software (supporting), writing – review and editing (supporting). **Dörte Harpke:** data curation (supporting), investigation (equal), methodology (equal), software (lead), writing – review and editing (supporting). **Oyuntsetseg Batlai:** data curation (supporting), methodology (supporting), resources (equal), writing – review and editing (equal). **Karsten Wesche:** conceptualization (lead), data curation (equal), formal analysis (equal), funding acquisition (lead), investigation (equal), methodology (equal), project administration (equal), resources (lead), supervision (lead), validation (lead), writing – review and editing (supporting). **Christiane M. Ritz:** conceptualization (lead), data curation (equal), funding acquisition (lead), investigation (equal), methodology (equal), project administration (lead), resources (lead), supervision (lead), validation (equal), writing – review and editing (supporting).

## Conflicts of Interest

The authors declare the following financial interests/personal relationships which may be considered as potential competing interests: Khurelpurev Oyundelger reports financial support was provided by TU Dresden. Karsten Wesche reports travel was provided by German Federal Ministry of Education and Research. Christiane Ritz reports travel was provided by German Academic Exchange Service.

## Supporting information


Table S1.
Table S2.
Table S3.
Table S4.
Table S5.
Table S6.
Table S7.
Table S8.
Table S9.
Table S10.
Table S11.
Table S12.

## Data Availability

WGS raw sequencing data are available in the NCBI Sequence Read Archive (SRA) under BioProject PRJNA680535. Further dataset generated and analyzed during the current study are provided in the Supplement material tables. The data that support the findings of this study are available on request from the corresponding author.

## References

[ece370419-bib-0001] Abràmoff, M. D. , P. J. Magalhães , and S. J. Ram . 2004. “Image Processing With ImageJ.” Biophotonics International 11: 36–42.

[ece370419-bib-0002] Al‐Ajmi, A. H. , M. S. Al‐Wahibi , A. E.‐Z. Mustafa , D. A. Soliman , and Y. H. Dewir . 2021. “Morphological and Molecular Assessment of Genetic Diversity of Seven Species of the Genus *Artemisia* L. (Asteraceae).” Arabian Journal for Science and Engineering 46: 5361–5371.

[ece370419-bib-0003] Amelchenko, V. P. 1979. “Contribution to Study of *Artemisia* From Yenisei Group.” *New Data on the Siberian Nature*. [Амельченко В.П. 1979. К изучению полыней Приенисейской группы // Новые данные о природе Сибири. Томск. С. 114–118], Tomsk, pp. 114–118.

[ece370419-bib-0004] Baasanmunkh, S. , M. Urgamal , B. Oyuntsetseg , et al. 2022. “Flora of Mongolia: Annotated Checklist of Native Vascular Plants.” PhytoKeys 192: 63–169. 35437387 10.3897/phytokeys.192.79702 PMC8938380

[ece370419-bib-0005] Balfourier, F. , G. Charmet , and C. Ravel . 1998. “Genetic Differentiation Within and Between Natural Populations of Perennial and Annual Ryegrass (*Lolium perenne* and *L. rigidum*).” Heredity 81: 100–110.

[ece370419-bib-0006] Brazier, T. , and S. Glémin . 2022. “Diversity and Determinants of Recombination Landscapes in Flowering Plants.” PLoS Genetics 18: e1010141. 36040927 10.1371/journal.pgen.1010141 PMC9467342

[ece370419-bib-0072] Bruvo, R. , N. K. Michiels , T. G. D’Souza , and H. Schulenburg . 2004. “A Simple Method for the Calculation of Microsatellite Genotype Distances Irrespective of Ploidy Level.” Molecular Ecology 13: 2101–2106. 15189230 10.1111/j.1365-294X.2004.02209.x

[ece370419-bib-0007] Bucher, S. F. , K. Auerswald , S. Tautenhahn , et al. 2016. “Inter‐and Intraspecific Variation in Stomatal Pore Area Index Along Elevational Gradients and Its Relation to Leaf Functional Traits.” Plant Ecology 217: 229–240.

[ece370419-bib-0008] Chevin, L.‐M. , and A. A. Hoffmann . 2017. “Evolution of Phenotypic Plasticity in Extreme Environments.” Philosophical Transactions of the Royal Society, B: Biological Sciences 372: 20160138. 10.1098/rstb.2016.0138 PMC5434089 28483868

[ece370419-bib-0009] Clark, L. V. , and M. Jasieniuk . 2011. “POLYSAT: An R Package for Polyploid Microsatellite Analysis.” Molecular Ecology Resources 11: 562–566. 21481215 10.1111/j.1755-0998.2011.02985.x

[ece370419-bib-0010] Csilléry, K. , O. Ovaskainen , C. Sperisen , N. Buchmann , A. Widmer , and F. Gugerli . 2020. “Adaptation to Local Climate in Multi‐Trait Space: Evidence From Silver Fir (*Abies alba* Mill.) Populations Across a Heterogeneous Environment.” Heredity 124: 77–92. 31182819 10.1038/s41437-019-0240-0 PMC6906498

[ece370419-bib-0011] De Kort, H. , J. G. Prunier , S. Ducatez , et al. 2021. “Life History, Climate and Biogeography Interactively Affect Worldwide Genetic Diversity of Plant and Animal Populations.” Nature Communications 12: 516. 10.1038/s41467-021-20958-2 PMC7822833 33483517

[ece370419-bib-0012] Dobson, A. J. , and A. G. Barnett . 2018. “Model fitting.” An Introduction to Generalized Linear Models, edited by C. Chatfield and J. Zidek , 25–49. Boca Raton, London, New York, Washington, DC: CRC Press Company.

[ece370419-bib-0013] Excoffier, L. , P. E. Smouse , and J. M. Quattro . 1992. “Analysis of Molecular Variance Inferred From Metric Distances Among DNA Haplotypes: Application to Human Mitochondrial DNA Restriction Data.” Genetics 131: 479–491. 1644282 10.1093/genetics/131.2.479 PMC1205020

[ece370419-bib-0014] Fick, S. E. , and R. J. Hijmans . 2017. “WorldClim 2: New 1‐km Spatial Resolution Climate Surfaces for Global Land Areas.” International Journal of Climatology 37: 4302–4315.

[ece370419-bib-0015] Garcia, S. , M. Sanz , T. Garnatje , A. Kreitschitz , E. D. McArthur , and J. Vallès . 2004. “Variation of DNA Amount in 47 Populations of the Subtribe Artemisiinae and Related Taxa (Asteraceae, Anthemideae): Karyological, Ecological, and Systematic Implications.” Genome 47: 1004–1014. 15644958 10.1139/g04-061

[ece370419-bib-0016] Gratani, L. 2014. “Plant Phenotypic Plasticity in Response to Environmental Factors.” Advances in Botany 2014: 208747.

[ece370419-bib-0017] Gupta, R. C. , H. Goyal , and V. Singh . 2014. “Cytology of the Genus *Artemisia* (Anthemidae, Asteraceae) in the Western Himalayas.” Biologia 69: 1134–1141.

[ece370419-bib-0018] Hamrick, J. L. , and M. J. Godt . 1990. “Allozyme Diversity in Plant Species.” In Plant Population Genetics, Breeding and Genetic Resources, edited by H. D. A. Brown , T. M. Clegg , L. A. Kahler , and S. B. Weir , 43–63. Sunderland, MA: Sinauer.

[ece370419-bib-0019] Hamrick, J. L. , and M. J. W. Godt . 1996. “Effects of Life History Traits on Genetic Diversity in Plant Species.” Philosophical Transactions of the Royal Society of London. Series B: Biological Sciences 351: 1291–1298.

[ece370419-bib-0020] Heelemann, S. , V. Bäuerlein , C. B. Krug , K. J. Esler , P. Poschlod , and C. Reisch . 2015. “Genetic Variation of Two Species With Different Life‐History Traits in the Endangered Renosterveld of South Africa—A Comparative Analysis of *Eriocephalus Africanus* and *Hemimeris racemosa* .” African Journal of Ecology 53: 447–453.

[ece370419-bib-0021] Hilbig, W. 1995. The Vegetation of Mongolia. Ulaanbaatar, Mongolia: SPB Academic Publishing.

[ece370419-bib-0022] Hughes, P. W. , W. J. J. Soppe , and M. C. Albani . 2019. “Seed Traits Are Pleiotropically Regulated by the Flowering Time Gene PERPETUAL FLOWERING 1 (PEP1) in the Perennial *Arabis alpina* .” Molecular Ecology 28: 1183–1201. 30712274 10.1111/mec.15034 PMC6850658

[ece370419-bib-0023] Hussain, A. , D. Potter , S. Kim , M. Q. Hayat , and S. A. I. Bokhari . 2019. “Molecular Phylogeny of *Artemisia* (Asteraceae‐Anthemideae) With Emphasis on Undescribed Taxa From Gilgit‐Baltistan (Pakistan) Based on nrDNA (ITS and ETS) and cpDNA (psbA‐trnH) Sequences.” Plant Ecology and Evolution 152: 507–520.

[ece370419-bib-0024] Jiao, J. , L. Han , Y. Jia , D. Lei , N. Wang , and L. Li . 2013. “Seed Morphology Characteristics in Relation to Seed Loss by Water Erosion in the Loess Plateau.” SpringerPlus 2: S9. 24701392 10.1186/2193-1801-2-S1-S9 PMC3973414

[ece370419-bib-0025] Kamvar, Z. N. , J. F. Tabima , and N. J. Grünwald . 2014. “Poppr: An R Package for Genetic Analysis of Populations With Clonal, Partially Clonal, and/or Sexual Reproduction.” PeerJ 2: e281. 24688859 10.7717/peerj.281 PMC3961149

[ece370419-bib-0026] Karbstein, K. , S. Tomasello , and K. Prinz . 2019. “Desert‐Like Badlands and Surrounding (Semi‐) Dry Grasslands of Central Germany Promote Small‐Scale Phenotypic and Genetic Differentiation in *Thymus praecox* .” Ecology and Evolution 9: 14066–14084. 31938504 10.1002/ece3.5844 PMC6953696

[ece370419-bib-0071] Karger, D. N. , O. Conrad , J. Böhner , et al. 2017. “Climatologies At High Resolution for the Earth's Land Surface Areas.” Scientific Data 4: 170122. 28872642 10.1038/sdata.2017.122 PMC5584396

[ece370419-bib-0027] Kawatani, T. 1964. “Chromosome Numbers in *Artemisia* .” Eisei Shikenjo Hōkoku 82: 183–193.

[ece370419-bib-0028] Knight, C. A. , and D. D. Ackerly . 2003. “Evolution and Plasticity of Photosynthetic Thermal Tolerance, Specific Leaf Area and Leaf Size: Congeneric Species From Desert and Coastal Environments.” New Phytologist 160: 337–347. 33832168 10.1046/j.1469-8137.2003.00880.x

[ece370419-bib-0030] Korobkov, A. A. , V. V. Kotseruba , and N. S. Probatova . 2014. “Chromosome Numbers of Some Species of *Artemisia* L. From Altai Region, South Siberia.” Botanica Pacifica: A Journal of Plant Science and Conservation 3: 61–66.

[ece370419-bib-0029] König, P. , S. Tautenhahn , J. H. C. Cornelissen , J. Kattge , G. Bönisch , and C. Römermann . 2018. “Advances in Flowering Phenology Across the Northern Hemisphere Are Explained by Functional Traits.” Global Ecology and Biogeography 27: 310–321.

[ece370419-bib-0031] Locascio, A. , M. Lucchin , and S. Varotto . 2009. “Characterization of a MADS FLOWERING LOCUS C‐LIKE (MFL) Sequence in *Cichorium intybus*: A Comparative Study of CiMFL and AtFLC Reveals Homologies and Divergences in Gene Function.” New Phytologist 182: 630–643. 19291007 10.1111/j.1469-8137.2009.02791.x

[ece370419-bib-0032] Matesanz, S. , and J. A. Ramírez‐Valiente . 2019. “A Review and Meta‐Analysis of Intraspecific Differences in Phenotypic Plasticity: Implications to Forecast Plant Responses to Climate Change.” Global Ecology and Biogeography 28: 1682–1694.

[ece370419-bib-0033] Meirmans, P. G. 2020. “Genodive Version 3.0: Easy‐To‐Use Software for the Analysis of Genetic Data of Diploids and Polyploids.” Molecular Ecology Resources 20: 1126–1131. 32061017 10.1111/1755-0998.13145 PMC7496249

[ece370419-bib-0034] Munkhzul, O. , K. Oyundelger , N. Narantuya , et al. 2021. “Grazing Effects on Mongolian Steppe Vegetation – A Systematic Review of Local Literature.” Frontiers in Ecology and Evolution 9: 703220.

[ece370419-bib-0035] Nybom, H. 2004. “Comparison of Different Nuclear DNA Markers for Estimating Intraspecific Genetic Diversity in Plants.” Molecular Ecology 13: 1143–1155. 15078452 10.1111/j.1365-294X.2004.02141.x

[ece370419-bib-0036] Nybom, H. , and I. V. Bartish . 2000. “Effects of Life History Traits and Sampling Strategies on Genetic Diversity Estimates Obtained With RAPD Markers in Plants.” Perspectives in Plant Ecology, Evolution and Systematics 3: 93–114.

[ece370419-bib-0037] Odat, N. , G. Jetschke , and F. H. Hellwig . 2004. “Genetic Diversity of *Ranunculus acris* L. (Ranunculaceae) Populations in Relation to Species Diversity and Habitat Type in Grassland Communities.” Molecular Ecology 13: 1251–1257. 15078460 10.1111/j.1365-294X.2004.02115.x

[ece370419-bib-0073] Oksanen, J. , R. Kindt , P. Legendre , et al. 2007. “The vegan Package.” Community Ecology Package 10: 631–637.

[ece370419-bib-0038] Ordoñez, J. C. , P. M. Van Bodegom , J. M. Witte , I. J. Wright , P. B. Reich , and R. Aerts . 2009. “A Global Study of Relationships Between Leaf Traits, Climate and Soil Measures of Nutrient Fertility.” Global Ecology and Biogeography 18: 137–149.

[ece370419-bib-0041] Oyundelger, K. , C. M. Ritz , O. Munkhzul , et al. 2020. “Climate and Land Use Affect Genetic Structure of *Stipa glareosa* PA Smirn. in Mongolia.” Flora 266: 151572.

[ece370419-bib-0040] Oyundelger, K. , O. Munkhzul , C. M. Ritz , and K. Wesche . 2023. “Long‐Term Grazing Exclusion Effects Populations Genetics and Functional Traits of *Artemisia frigida* in Mongolia.” Journal of Arid Environments 209: 104900.

[ece370419-bib-0039] Oyundelger, K. , V. Herklotz , D. Harpke , B. Oyuntsetseg , K. Wesche , and C. Ritz . 2021. “Contrasting Effects of Local Environment and Grazing Pressure on Genetic Diversity and Structure of *Artemisia frigida* .” Conservation Genetics 22: 947–962.

[ece370419-bib-0042] Pan, S. , C. Liu , W. Zhang , et al. 2013. “The Scaling Relationships Between Leaf Mass and Leaf Area of Vascular Plant Species Change With Altitude.” PLoS One 8: e76872. 24146938 10.1371/journal.pone.0076872 PMC3795618

[ece370419-bib-0043] Paradis, E. , and K. Schliep . 2019. “Ape 5.0: An Environment for Modern Phylogenetics and Evolutionary Analyses in R.” Bioinformatics 35: 526–528. 30016406 10.1093/bioinformatics/bty633

[ece370419-bib-0044] Pellicer, J. , S. Garcia , M. A. Canela , et al. 2010. “Genome Size Dynamics in *Artemisia* L. (Asteraceae): Following the Track of Polyploidy.” Plant Biology 12: 820–830. 20701707 10.1111/j.1438-8677.2009.00268.x

[ece370419-bib-0045] Perez‐Harguindeguy, N. , S. Diaz , E. Garnier , et al. 2013. “New Handbook for Standardised Measurement of Plant Functional Traits Worldwide.” Australian Journal of Botany 61: 167–234.

[ece370419-bib-0046] R Core Team . 2020. R: A Language and Environment for Statistical Computing. Vienna: R Foundation for Statistical Computing.

[ece370419-bib-0047] Reisch, C. , and M. Bernhardt‐Römermann . 2014. “The Impact of Study Design and Life History Traits on Genetic Variation of Plants Determined With AFLPs.” Plant Ecology 215: 1493–1511.

[ece370419-bib-0048] Riggins, C. W. , and D. S. Seigler . 2012. “The Genus *Artemisia* (Asteraceae: Anthemideae) at a Continental Crossroads: Molecular Insights Into Migrations, Disjunctions, and Reticulations Among Old and New World Species From a Beringian Perspective.” Molecular Phylogenetics and Evolution 64: 471–490. 22580463 10.1016/j.ympev.2012.05.003

[ece370419-bib-0049] Rosenberg, N. A. 2004. “DISTRUCT: A Program for the Graphical Display of Population Structure.” Molecular Ecology Notes 4: 137–138.

[ece370419-bib-0050] Sanz, M. , G. Schneeweiss , R. Vilatersana Lluch , and J. Vallès Xirau . 2011. “Temporal Origins and Diversification of *Artemisia* and Allies (Anthemideae, Asteraceae).” Collectanea Botanica 30: 7–15.

[ece370419-bib-0070] Sanz, M. , R. Vilatersana , O. Hidalgo , et al. 2008. “Molecular Phylogeny and Evolution of Floral Characters of *Artemisia* and Allies (Anthemideae, Asteraceae): Evidence From nrDNA ETS and ITS Sequences.” Taxon 57: 66–78.

[ece370419-bib-0051] Scheepens, J. F. , E. S. Frei , and J. Stöcklin . 2010. “Genotypic and Environmental Variation in Specific Leaf Area in a Widespread Alpine Plant After Transplantation to Different Altitudes.” Oecologia 164: 141–150. 20461412 10.1007/s00442-010-1650-0

[ece370419-bib-0052] Stotz, G. C. , C. Salgado‐Luarte , V. M. Escobedo , F. Valladares , and E. Gianoli . 2022. “Phenotypic Plasticity and the Leaf Economics Spectrum: Plasticity Is Positively Associated With Specific Leaf Area.” Oikos 2022: e09342.

[ece370419-bib-0053] Szczecińska, M. , G. Sramko , K. Wołosz , and J. Sawicki . 2016. “Genetic Diversity and Population Structure of the Rare and Endangered Plant Species *Pulsatilla patens* (L.) Mill in East Central Europe.” PLoS One 11: e0151730. 27003296 10.1371/journal.pone.0151730 PMC4803199

[ece370419-bib-0054] Tkach, N. V. , M. H. Hoffmann , M. Röser , A. A. Korobkov , and K. B. Von Hagen . 2008. “Parallel Evolutionary Patterns in Multiple Lineages of Arctic *Artemisia* L. (Asteraceae).” Evolution 62: 184–198. 17976192 10.1111/j.1558-5646.2007.00270.x

[ece370419-bib-0055] Tuvshintogtokh, I. 2014. The Steppe Vegetation of Mongolia. Edited by C. Sanchir , 610. Ulaanbaatar: Bembi san.

[ece370419-bib-0057] Vallès, J. , and E. D. McArthur . 2001. “ *Artemisia* Systematics and Phylogeny: Cytogenetic and Molecular Insights.” USDA Forest Service Proceedings 21: 67–74.

[ece370419-bib-0056] Vallès, J. , S. Garcia , O. Hidalgo , et al. 2011. “Biology, Genome Evolution, Biotechnological Issues and Research Including Applied Perspectives in *Artemisia* (Asteraceae).” In Advances in Botanical Research, edited by J.‐C. Kader and M. Delseny , 349–419. United States: Academic Press.

[ece370419-bib-0060] von Wehrden, H. , and K. Wesche . 2007. “Relationships Between Climate, Productivity and Vegetation in Southern Mongolian Drylands.” Basic and Applied Dryland Research 1: 100–120. 22318349 10.1127/badr/1/2007/100 PMC3272425

[ece370419-bib-0059] von Wehrden, H. , J. Hanspach , K. Ronnenberg , and K. Wesche . 2010. “Inter‐Annual Rainfall Variability in Central Asia—A Contribution to the Discussion on the Importance of Environmental Stochasticity in Drylands.” Journal of Arid Environments 74: 1212–1215.

[ece370419-bib-0058] von Wehrden, H. , J. Hanspach , P. Kaczensky , et al. 2012. “Global Assessment of the Non‐Equilibrium Concept in Rangelands.” Ecological Applications 22: 393–399. 22611842 10.1890/11-0802.1

[ece370419-bib-0061] Waitt, D. E. , and D. A. Levin . 1998. “Genetic and Phenotypic Correlations in Plants: A Botanical Test of Cheverud's Conjecture.” Heredity 80: 310–319.

[ece370419-bib-0062] Wang, Z. 2011. “Study on Genetic Diversity of Traditional Mongolian Medicine *Artemisia frigida*.” Master thesis, Minzu University of China.

[ece370419-bib-0063] Wang, Z. , S. Lv , G. Han , et al. 2022. “Heavy Grazing Reduced the Spatial Heterogeneity of *Artemisia frigida* in Desert Steppe.” BMC Plant Biology 22: 337. 35831803 10.1186/s12870-022-03712-8 PMC9281028

[ece370419-bib-0064] Wei, T. , V. R. Simko , M. Levy , Y. Xie , Y. Jin , and J. Zemla . 2021. “Package “Corrplot”: Visualization of a Correlation Matrix.” Version 0.84.

[ece370419-bib-0065] Wickham, H. 2011. “ggplot2.” Wiley Interdisciplinary Reviews: Computational Statistics 3: 180–185.

[ece370419-bib-0066] Woodward, F. I. 1983. “The Significance of Interspecific Differences in Specific Leaf Area to the Growth of Selected Herbaceous Species From Different Altitudes.” New Phytologist 95: 313–323.

[ece370419-bib-0067] Yi, F. , Z. Wang , C. C. Baskin , et al. 2019. “Seed Germination Responses to Seasonal Temperature and Drought Stress Are Species‐Specific but not Related to Seed Size in a Desert Steppe: Implications for Effect of Climate Change on Community Structure.” Ecology and Evolution 9: 2149–2159. 30847100 10.1002/ece3.4909 PMC6392344

[ece370419-bib-0068] Yulin, L. I. , D. A. Johnson , S. U. Yongzhong , C. U. I. Jianyuan , and T. Zhang . 2005. “Specific Leaf Area and Leaf Dry Matter Content of Plants Growing in Sand Dunes.” Botanical Bulletin of Academia Sinica 46.

[ece370419-bib-0069] Zhou, H.‐F. , X.‐M. Zheng , R.‐X. Wei , G. Second , D. A. Vaughan , and S. Ge . 2008. “Contrasting Population Genetic Structure and Gene Flow Between *Oryza rufipogon* and *Oryza nivara* .” Theoretical and Applied Genetics 117: 1181–1189. 18712516 10.1007/s00122-008-0855-7

